# Extracellular Vesicles: Interplay with the Extracellular Matrix and Modulated Cell Responses

**DOI:** 10.3390/ijms23063389

**Published:** 2022-03-21

**Authors:** Aleen Al Halawani, Suzanne M. Mithieux, Giselle C. Yeo, Elham Hosseini-Beheshti, Anthony S. Weiss

**Affiliations:** 1 Charles Perkins Centre, The University of Sydney, Camperdown, NSW 2006, Australia; aleen.alhalawani@sydney.edu.au (A.A.H.); suzanne.mithieux@sydney.edu.au (S.M.M.); giselle.yeo@sydney.edu.au (G.C.Y.); 2 School of Life and Environmental Sciences, The University of Sydney, Camperdown, NSW 2006, Australia; 3 School of Medical Sciences, Faculty of Medicine and Health, The University of Sydney, Camperdown, NSW 2006, Australia; elham.beheshti@sydney.edu.au; 4 Sydney Nano Institute, The University of Sydney, Camperdown, NSW 2006, Australia

**Keywords:** extracellular vesicles, exosomes, microvesicles, extracellular matrix, mesenchymal stem cells, wound healing, tissue repair, matrix-bound vesicles

## Abstract

The discovery that cells secrete extracellular vesicles (EVs), which carry a variety of regulatory proteins, nucleic acids, and lipids, has shed light on the sophisticated manner by which cells can communicate and accordingly function. The bioactivity of EVs is not only defined by their internal content, but also through their surface associated molecules, and the linked downstream signaling effects they elicit in target cells. The extracellular matrix (ECM) contains signaling and structural molecules that are central to tissue maintenance and repair. Recently, a subset of EVs residing within the extracellular matrix has been identified. Although some roles have been proposed for matrix-bound vesicles, their role as signaling molecules within the ECM is yet to be explored. Given the close association of EVs and the ECM, it is not surprising that EVs partly mediate repair and regeneration by modulating matrix deposition and degradation through their cellular targets. This review addresses unique EV features that allow them to interact with and navigate through the ECM, describes how their release and content is influenced by the ECM, and emphasizes the emerging role of stem-cell derived EVs in tissue repair and regeneration through their matrix-modulating properties.

## 1. Introduction

Extracellular vesicles (EVs) are generated by most cells as part of their normal physiology and released into the extracellular environment to maintain tissue homeostasis. Although they are delimited by a lipid membrane bilayer and share some surface features [1], the content, biogenesis, and release of EVs vary depending on their cellular origin, metabolic state, and the surrounding environment [2,3]. As parent cell content and thus information is packed into EVs, they reflect the state of the parent cell [4]. This feature makes them fit for use as biomarkers for injuries or pathologies [5,6,7,8]. From a physiological perspective, EVs from injured cells contribute towards tissue repair by facilitating intercellular communication between affected cells and their surroundings. This is exemplified by stem cells and immune cells, which upon exposure to stress are induced to release EVs [1,2,9].

Broadly, EVs can be categorized into large EVs (<5000 nm) which originate from the plasma membrane (apoptotic bodies and microvesicles/ectosomes) [3,10] and small EVs (<150 nm) derived from endosomal compartments (exosomes) [3,11]. Despite efforts to separate these two subclasses, some of their overlapping features and similar roles in transferring molecular cargo pose a challenge to achieving a clear distinction between EV specific and general functional characteristics [11]. Due to EV heterogeneity and current limitations in EV characterization, most published studies utilize microvesicles and exosomes. Although apoptotic bodies could also be implicated, their larger size means that they are mostly excluded due to sample preparation measures. EVs from biological fluids or from in-vitro-conditioned cell cultures can be exploited for various translational purposes, including the diagnosis and prognosis of different pathological disorders [12] or as mediators of tissue repair [13] through angiogenesis, inhibition of apoptosis, promotion of cell proliferation [14], and modulating matrix synthesis [9,15,16].

While it is conventionally accepted that EVs are secreted into biological fluids which enables their free shuttling between cells, in tissues EVs inevitably encounter the ECM and therefore must be able to traverse it. This is manifested in either an active manner, such as that exhibited by cancer derived EVs, or in a more passive manner that seems to depend on the combined properties of EVs and ECM [17]. The ECM as a supporting environment facilitates EV transport and influences EV content and release. Furthermore, the discovery that matrix-bound vesicles (which are a subpopulation of EVs) [18,19,20] are specifically resident in the ECM suggests that EVs may confer signaling cues within the extracellular environment [18,19]. Collectively, these findings indicate that the ECM is more than a passive conveying medium that EVs simply need to traverse. This review explores how EVs are transported through and influenced by the ECM, and how they modulate the ECM either through the molecules they carry or through functional changes they elicit in their targets, ultimately contributing to tissue repair.

## 2. Biogenesis of EVs

**Figure 1:**
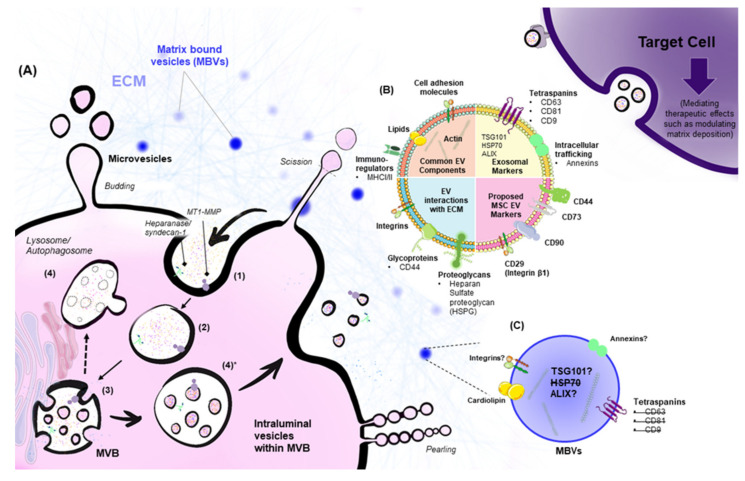
Extracellular Vesicle biogenesis and features. (**A**) Microvesicles or ectosomes form via outward budding of the plasma membrane, pearling, and subsequent scission. (1) Endocytosis of extracellular components results in the formation of an early sorting endosome. Extracellular matrix proteins such as syndecan and other transmembrane proteins such as MT1-MMP can also be endocytosed, and subsequently associate with forming intraluminal vesicles [27]. The early endosome (2) then matures into a late sorting endosome that exchanges its cargo with intracellular constituents such as the trans-Golgi network and the endoplasmic reticulum. (3) The involution of the membrane of the endosome (late sorting endosome) results in the formation of intraluminal vesicles. A multivesicular body with multiple intraluminal vesicles faces three fates: (4) starvation, followed by fusion with autophagosome, fusion with lysosomes, or (4)* docking onto the plasma membrane and fusing to release contents as exosomes. Following their release, EVs traverse the ECM to interact with target cells or are transported in biological fluids. EV-target-cell interactions take place through various mechanisms. Entry of intact exosomes can occur via receptor-mediated endocytosis, phagocytosis, micropinocytosis, clathrin-mediated entry, caveolae, or lipid-raft-mediated endocytosis [3,28]. Alternatively, exosomes can fuse directly with their target cells, or mediate intracellular signaling by direct-receptor binding. After they interact with target cells, EVs can mediate therapeutic effects that involve ECM protein regulation. Common exosomal markers and EV properties are outlined in (**B**), along with EV components that interact with the ECM. Proposed EV MSC markers are outlined [29]. There is a subpopulation of EVs within the ECM (MBVs). MBVs are enriched in actin and cardiolipin, but it is not clear whether they are enriched in other common exosomal or microvesicle markers such as Annexin A1 [30]. As depicted in (**C**), MBVs do not express key exosomal markers; these absent markers are struck out with a line. The figure is adapted from [3,9,21]. MT1-MMP: Membrane-type matrix metalloproteinase; EVs: Extracellular vesicles; ECM: Extracellular matrix; MSC: Mesenchymal stem cell; MBVs: Matrix-bound vesicles.

There are three general mechanisms that govern EV formation: apoptosis, ectosomal shedding, and exosome biogenesis via endosomal pathways [3]. Apoptotic bodies range in diameter from 500–5000 nm, and form when cells compartmentalize their content and undergo programmed cell death [10]. Ectosomes, also known as microvesicles (Figure 1A), are generated through outward budding of the cell plasma membrane, producing vesicles ranging from 50–1000 nm in diameter [3]. Inherent cellular protrusions such as microvilli, as well as other types of protrusions observed during cell division and migration such as filopodia and retraction fibers, are also linked to ectosome biogenesis and act as a source of EVs [21,22].

Budding entails plasma membrane protrusion from the cytosol. Additionally, newly-formed or inherent cellular protrusions can undergo a pearling process, followed by subsequent scission and release. These processes refer to different mechanisms of ectosomal shedding, however, they usually imply the same outcome; the formation of ectosomes/microvesicles. All these mechanisms involve the lipid bilayer and cytoskeletal structures such as actin; however, on rare occasions actin is not present, particularly in vesicles shedding from the distal tips of actin-based projections [23,24]. Pearling refers to sequential vesicles forming from a cellular protrusion such as a microvillus, with an appearance that is analogous to pearls on a string. Pearling is usually observed as a trail of aligned vesicles. Although the mechanisms resulting in pearling are not understood, it may be a membrane-driven process because cholesterol depletion appears to increase pearling and subsequent vesicular release [25], most likely due to compromised membrane integrity. Additionally, giant unilamellar vesicles, which mimic the behavior of cell membranes, exhibit transient pearling upon exposure to a negative osmotic gradient [26]. Scission generally refers to the protein-induced process of cleaving membrane-derived vesicles, including those released from the distal tips of elongated cellular protrusions such as filopodia [22]. Budding and shedding are usually used in conjunction to refer to the process of plasma membrane protrusion, followed by narrowing at the base of the protrusion and subsequent scission.

Exosomes differ from apoptotic bodies and microvesicles in that they originate from endosomal pathways that are traditionally involved in endocytic recycling or lysosomal degradation (Figure 1A,B). Endocytosis of lipids, proteins, or other metabolites within the extracellular environment generates early sorting endosomes (ESEs). Because endosomes are derived from the plasma membrane, they include cell-surface entities such as trans-membrane and soluble proteins, but the outer lipid membrane leaflet is on the inside of the organelle/compartment, and thus the inner membrane leaflet is on the outside. ESEs then mature into late sorting endosomes (LSEs) that generate multivesicular bodies (MVBs) which house multiple intraluminal vesicles (ILVs) ranging in diameter from 40–160 nm [3]. ILVs are generated upon a second invagination of the MVBs so that the outer-membrane leaflet is restored to its original position on the outside. MVBs either fuse with the plasma membrane and release the intraluminal vesicles as exosomes, or they fuse with lysosomes/autophagosomes and their content is degraded.

## 3. Signaling and Metabolic Properties

While the mechanisms governing EV biogenesis differ, as described above, the range of initial interactions with downstream target cells are similar. When EVs are released into the extracellular space, they either flow freely [2] or interact with the extracellular environment to reach their target [27]. Upon reaching their target, they broadly mediate their effects through cell-signaling cascades initiated by EV-receptor interactions [3], and/or by transferring their contents to the cells with which they fuse or by which they are internalized [3]. Fusion of EVs with target cells can transfer receptors on the EV surface to the recipient cell plasma membrane [31], as well as RNA, DNA, lipids, and proteins [32]. RNA subtypes carried by EVs are of particular interest due to their ability to alter target cell gene expression and serve as templates for protein synthesis. This latter population includes coding RNA in the form of fragmented and intact mRNA. Non-coding RNA content comprises a mix of long non-coding RNA (lncRNA), ribosomal RNA (rRNA), and small non-coding RNA such as miRNA, siRNA and piRNA [33].

Although therole of EVs in intercellular communication has been appreciated, it has also been proposed that they can act as independent metabolic entities that influence their surrounding microenvironment. This has been demonstrated by taking EVs that were isolated from media conditioned by neural stem cells, and incubating these in fresh media while monitoring metabolite consumption and release. Aspartate and glutamate were among the most abundantly released metabolites, whereas asparagine was the most consumed metabolite, suggesting high levels of asparaginase activity [34]. Additionally, other studies investigating MSC exosomal proteome revealed five key enzymes: GAPDH, PGK, PGM, ENO, PKM2 all of which are involved in glycolysis [35].

## 4. EVs and Matrix Interactions

### 4.1. Active Transport

In addition to these biochemical properties, EVs play an active role in matrix remodeling through surface molecular interactions or the downstream effector roles of their cargo [27,36]. The enzyme-like functions exhibited by EVs under physiological conditions or in wound healing are not well understood. However, evidence from cancer-derived EVs suggests that proteases and glycosidases on the EV surface contribute to matrix remodeling and degradation, in addition to growth factor mobilization from the ECM [36].

Transmembrane proteoglycans with heparan sulfate chains, such as syndecan-1, associate with and can be attacked by heparanase. EV biogenesis pathways are known to retain heparanase on the surface of released EVs, and upon exposure to ECM, heparanase degrades their heparan sulfate components and liberates associated signaling molecules, as demonstrated in exosomes derived from myeloma, lymphoblastoid and breast cancer cell lines [36,37,38]. Glypican, another cell-surface heparan-sulfate proteoglycan (HSPG), is internalized from the cell surface and associates with exosomes in a similar manner, while HSPGs in target cells are essential for exosome internalization by recipient cells [39]. This was demonstrated by co-localization of internalized exosomes with fluorescently tagged syndecan and glypican. Furthermore, exosome internalization was impaired upon treating recipient cells with heparinase. Other cell-surface enzymes that associate with exosomes due to the nature of their biogenesis include enzymes with transmembrane domains, such as MT1-MMP [36,40] which is important in angiogenesis and wound healing. MT1-MMP degrades fibrillar collagens and other matrix proteins such as fibronectin, or activates MMP-2, which exhibits similar matrix-degrading properties. The association of matrix components and matrix-degrading molecules on the EV surface not only supports a role for EVs in actively interacting with and escaping matrix confinement via degrading matrix proteins, but also provides a mechanism for EV uptake by recipient cells [39].

Unlike EVs from cancer sources in which these surface enzymes were identified and explored, stem-cell-derived EVs have not been characterized by surface functionality. It is not clear whether enzymes identified in cancer correlate with those under normal physiological conditions. Interrogation of Exocarta (an online database of exosomal content in different species and cell types/sources) (exocarta.org) indicates that >100 matrix-related proteins are linked to mesenchymal stem-cell-derived exosomes, contributing to 12% of the total protein entries for mesenchymal stem cell exosomes. However, neither clear description nor further validation of these entries has been reported in the literature. Nevertheless, there is emerging evidence demonstrating the downstream functional and structural changes in the ECM spurred by stem-cell-derived-EVs [4,13,14,41,42,43,44,45,46].

### 4.2. Passive Transport

EVs can passively navigate the ECM. While the porosity of the ECM (<50 nm) is usually not large enough to allow for passive EV transport and diffusion, a recent study demonstrated a comprehensive mechanism in which matrix mechanics, in conjunction with the fluctuating deformation of EVs, contribute to EV transport. EVs in stiff stress-relaxing systems escape confinement more rapidly than EVs in soft stress-relaxing systems. Stress-relaxation refers to a gradual decrease in stress over time upon the application of constant strain, and mostly applies to viscoelastic materials [47]. For example, when the ECM is held at constant strain, less stress will be required over time to maintain that strain, essentially expediting EV transport. In contrast, in elastic systems, applied stress must be maintained to maintain strain. In addition to the stress-relaxation properties of the ECM, the fluctuating deformation of EVs due to water permeation via aquaporin-1, a feature lacking in liposomes and synthetic nanoparticles, further supports EV transport [17]. To eliminate the influence of biological and biochemical interactions on EV transport, inert alginate-based hydrogels were utilized as ECM-mimicking platforms. These hydrogels were used to assess EV transport through the ECM by quantifying EV release from these systems. EV release from hydrogels was not due to matrix degradation, neither was it affected by the absence of calcium, which plays a key role in cytoskeletal contractility that could contribute to EV deformation. Instead, EV release appeared to depend on the hydrogel’s mechanics. In the presence of stiff stress-relaxing hydrogels, EV transport and release were better than in stiff elastic hydrogels. Stress-relaxing systems differ from elastic systems in their ability to dissipate stress [48]. These observations are consistent among EVs derived from different cell types, implying that fluctuating deformation facilitated by ECM mechanical properties is an inherent property of EVs [17].

### 4.3. Effect of the ECM on EV Content and Release

Additionally, matrix composition and consequent changes in its mechanical properties affect EV release and content. For example, an early study revealed that stress-relaxation of fibroblasts triggered an ectocytic process of transient membrane budding that resulted in the release of small plasma membrane derived vesicles into the collagen matrix in which they were cultured [49]. In another study, MSCs cultured on soft hydrogels with low ligand density for integrin interaction were found to release more EVs than those cultured on stiff hydrogels or tissue-culture plastic [50]. These findings indicate that EV release, not just its transport, is influenced by physical properties of the ECM, such as viscoelasticity and the availability of sites for biological interactions.

Whether these features alter the content of EVs is unclear. A study into the effect of fibrosis on EV content and bioactivity showed that lung stiffness due to fibrosis increases pro-fibrotic miRNA (miR-21) content within the EVs, which further accentuates fibrosis by lung-derived fibroblasts. EVs derived from fibrotic rat lung fibrocytes, which are bone-marrow-derived cells expressing hematopoietic and stromal cell markers [51], significantly increased *COL1A1* expression in normal fibroblasts, which is indicative of scarring. Furthermore, higher levels of miR-21-5p, which is associated with fibrosis, have been noted within EVs from fibrocytes that were cultured on rigid plates and on decellularized fibrotic lung scaffolds, compared to those cultured on soft plates and non-fibrotic lung scaffolds, respectively [52]. These findings support the suggestion that mechanical and biological changes in the ECM are ultimately reflected in released EVs. Fibrocyte-derived EVs carrying miR-21-5p were found to be taken up by lung fibroblasts more frequently than were EVs released by the lung fibroblasts themselves, as demonstrated by tracking and flow cytometry studies. Taken together, these data support the concept that abnormalities in the ECM, such as those that occur in pulmonary fibrosis, are amplified in a self-promoting cycle that involves EVs. Further studies investigating the functional changes of EVs arising from alterations in the ECM can be used to inform the design of matrix-mimicking hydrogels and biomaterials in the development of scaffolds that do not adversely influence EV content and compromise intercellular communication.

### 4.4. EVs as Cues within the ECM

Although EVs play a role in ECM modulation by regulating matrix degradation/synthesis by target cells, it is largely unknown whether they continuously and actively regulate the ECM as intrinsic cues. Functional roles of EVs released into native ECM have not been reported in this context; however, matrix-bound vesicles (MBVs) have been seen in decellularized tissue from multiple sources and in decellularized scaffolds [17,18,19]. Interestingly, MBVs do not express canonical exosomal markers (CD63, CD81, CD9, and Hsp70) (Figure 1C). On treating macrophages with isolated MBVs, Fizz-1 marker expression increases, consistent with IL-4 macrophage stimulation and polarization towards an M2 phenotype. Treating macrophages with solubilized matrix from the same source as the MBVs elicits a similar expression pattern. These studies implicate MBVs as a potentially distinct population of signaling vesicles that affect cell behavior in a manner similar to that of their parent matrix.

To expand on these findings, a 2020 study aimed to distinguish between the two putative EV populations: EVs in biological fluids and matrix-bound vesicles. Two systems were used to corroborate previous observations of matrix-specific vesicles [19]. Decellularized and lyophilized porcine-urinary-bladder-derived-matrix was employed to visualize the localization of spherical bodies. Due to the heterogeneity of EV sources within native ECM, 3T3 fibroblasts were used as a model system; cells were grown in the presence of ascorbic acid to induce matrix deposition. After seven days, conditioned media were harvested and the deposited matrix was obtained by decellularization, then MBVs were obtained by enzyme digestion of the ECM. In line with previous findings [18], traditional EV markers were lower in matrix-derived EVs compared with liquid-phase EVs. There were marked differences between the protein cargo in the two EV populations, and an enrichment of small RNAs, with a broader distribution of small RNAs in liquid-phase EVs. There were 28 differentially expressed miRNAs in the matrix population, including miR-163-5, miR27a-5p, miR-99-5p, compared with the liquid phase population. Ingenuity pathway analysis (IPA) revealed that miRNAs associated with MBVs were linked to organ development while those enriched in the liquid phase were associated with cell growth, development, and proliferation pathways. This IPA implies that MBVs contributed to tissue homeostasis. Indeed, stem cell EVs host a range of morphogens and stem cell differentiation factors such as Wnt, Notch and Hh that may participate in bidirectional communication between stem and parenchymal cells to activate and promote the differentiation of stem cells [2].

Bone marrow mesenchymal stem cells (BM-MSCs), adipose derived stem cells (ADSCs) and umbilical cord stem cells were used in a manner similar to that described above for fibroblasts, to establish that miRNA content is unique to cellular origin. Separate clustering of the generated miRNA libraries demonstrated clear differences in the miRNA profiles of EVs from the three different cell types [19]. In addition to miRNA content, phospholipid content also varied between the two EV populations, with cardiolipin notably lower in liquid phase EVs and in the parent cells. As cardiolipin is derived from the inner mitochondrial membrane, this matrix vesicle biogenesis appears to involve the mitochondrial compartment. It is clear from these studies that EVs associated with the ECM broadly contribute to a sub-population of EVs and that these could be used to engineer inductive scaffolds that promote regeneration by restoring bidirectional communication between stem cells and neighboring parenchyma.

## 5. EVs and Tissue Repair via Matrix Modulation

EVs isolated from liquids such as blood plasma and cell culture media are not specific to the ECM but have the capacity to mediate repair by activating signaling pathways that contribute to matrix modulation [41,42,43,44,45]. As an example, multiple studies have reported angiogenic roles pertaining to EVs [53], although the link between EV-angiogenic and matrix-modulatory roles in the context of regeneration is not clear.

### 5.1. ECM-Related Cargo

The secretomes of ADSCs and BM-MSCs have been widely studied for their regenerative potential, which can be powerful because they approximate the regenerative action of their parent cells. Some of the regenerative effects that are imparted by the secretome have been attributed to purified EV fractions from these cells [54]. Furthermore, it has been shown that ADSC EVs derived from porcine abdominal fat tissue are particularly enriched with glycoproteins and extracellular matrix proteins [55]. The human ADSC secretome contributes to extracellular matrix organization (FBLN2, TNXB and ABI3BP), and glycosaminoglycan metabolic (ITIH1, ITIH2, ITIH3) and catabolic (IDUA) processes, but these have been attributed broadly to conditioned media [56] and there are no differences in ECM-associated proteins between EVs and the conditioned media. Given that in this work the EVs were not pelleted from the conditioned media prior to concentration using 3 kDa MCO filters, it is quite possible that removing EVs from the conditioned media and then comparing EV content with EV-free conditioned media will shed light on their functionality.

### 5.2. EVs Mediate ECM Repair through Modulation by Target Cells

ADSC EVs play a potent role in the induction of matrix protein synthesis. This is underpinned by the finding that smooth muscle cells (SMCs) increased elastin and collagen deposition after 30 days of culture on 3D fibrin gel constructs when stimulated with EVs, compared to non-conditioned/control media and EV-depleted conditioned media. However, this was not strongly reflected in transcripts of genes typically involved in elastin fiber formation and maturation, such as tropoelastin (*ELN*), *FBLN4*, *FBLN5*, *LOX*, *LOXL*, and *LTBP-4*, as only fibrillin-1 expression increased after 30 days; the authors concluded that over 30 days, cells might have adapted to the stimulatory effect of EVs [41]. Likewise, BM-MSC EVs showed attenuated proteolytic activity in aneurysmal aorta SMC explants by reducing MMP-2 expression, paired with elevated TIMP-1 and TIMP-2. After 21 days in culture, *ELN*, *FBLN-5* and *LOX* gene expression increased in the EV-treated cells, compared to conditioned media controls (total media and without EVs) and this was coupled with dense elastic matrix deposition, which served to highlight the anti-proteolytic roles of such stem cell-derived EVs [43].

More recently, ADSC EVs were found to display therapeutic potential for attenuating osteoarthritis, partly through maintaining the chondrocyte-populated matrix and protecting cartilage from degeneration. These EVs decreased MMP-1, MMP-13 and ADAMTS-5 expression in human chondrocytes in vitro, and osteoarthritic cells in the presence of IL-1β [44,57]. Furthermore, EVs isolated from the conditioned media of an immortalized embryonic stem cell line promoted type II collagen deposition and healing of critical size osteochondral defects by as early as 2 weeks post-treatment. This was accompanied by elevated sulfated glycosaminoglycan levels and transcript levels of *COL2A1*, cartilage oligomeric matrix protein (*COMP*), and TGF-β [45]. Collectively, these findings help establish the idea that stem cell derived-EVs contribute to regeneration, at least in part through matrix modulation.

A mouse in vivo model of wound healing and repair revealed reduced scar size in full-thickness dorsal wounds treated with ADSC EVs. Collagen III levels were elevated at the wound site while collagen I levels were decreased [42]. Cutaneous healing benefits have also been proposed for EVs derived from BM-MSCs presenting a dense coat of hyaluronan, which participates in matrix remodeling, along with the hyaluronic acid receptor CD44 which is implicated in stem cell mobilization [58,59,60].

Vascularization of injured tissue is crucial in maintaining tissue survival and reducing scarring. EVs isolated from umbilical cord blood plasma improve wound closure, re-epithelialization, and endothelialization, contemporaneous with enhanced vascularization [46]. Fibroblast proliferation assays have been used to show enhanced proliferation in the presence of EVs, while endothelial tube formation assays parallel pro-angiogenic effects observed in vivo. It was proposed that EVs shuttle miR-21-3p into both fibroblasts and endothelial cells. Consequently, qRT-PCR analysis of cells stimulated with EVs revealed a marked increase in miR 21-3p, with reduced levels of miR 21-3p target genes, *PTEN* and *SPRY-1*. These effects were reversed upon inhibiting miR-21-3p, which implicates miR 21-3p in a targeting, functional or regulatory role. Studies on EVs obtained from umbilical cord blood plasma, while not widely investigated, show promise in wound healing similar to stem-cell-derived EVs (Table 1).

## 6. Conclusions

EVs from diverse sources share some similarities but ultimately can elicit different responses, primarily because they reflect the functionality of their source cells. The existence of ECM-specific EVs leads to the proposal that they play an active role in tissue homeostasis. This concept is further supported by the presence of EVs in explanted decellularized tissues, where they can contribute to regenerative effects, presumably by recapitulating aspects of missing cell–cell signaling in these scaffolds. The wound healing benefits of EVs can be attributed to their modulation of proteolysis in matrix remodeling but the effectors for this modulation are unknown. Stem cell EVs are of substantial interest due to their angiogenic and matrix-modulatory roles, which collectively contribute to improved healing. Although numerous studies have investigated matrix-modulatory and angiogenic roles separately, an understanding of the interactions between these two important aspects of the healing response will deliver improved repair by EVs. Stem cell derived EVs will benefit from more characterization as this knowledge will allow for the selection of matched EVs for specific outcomes, and should overcome current translational limitations in ECM repair.

## Figures and Tables

**Table 1:** Origin, cargo/markers, target, and downstream reparative effects of EVs.

EV Origin	Cargo or Markers	Target	Downstream Effects	Reference
Embryonic SC-derived MSC line	N/A	Osteochondral defect	↑ Collagen deposition ↑ Sulfated GAGs ↑ COL2A1 ↑ COMP ↑ TGF-β	[45]
ADSCs	Glycoproteins ECM proteins	Smooth muscle cells (SMCs)	Improved elastin and collagen deposition ↑ Fibrilin-1	[41]
ADSCs	N/A	Human chondrocyte osteoarthritis cells (HC-OA)	↓MMP-1 ↓ MMP-13 ↓ ADAMTS-5	[44]
ADSCs	N/A	Full thickness dorsal wound	↓ COL1A1 ↑ COL3A1	[42]
BM-MSCs	N/A	SMCs	↓MMP-2 ↑ TIMP-1, 2 ↑ ELN, FBLN-5 and LOX	[43]
Umbilical Cord Blood	miR 21-3p	Fibroblasts Endothelial cells	↑ miR 21-3p Improved cell proliferation and angiogenesis	[46]

N/A: Not available; ↑ increased/upregulated; ↓ decreased/downregulated; ADSCs: Adipose-derived stem cells; BM-MSCs: Bone marrow-derived mesenchymal stem cells; SC: stem cell; SMCs: Smooth muscle cells; HC-OA: Human chondrocyte osteoarthritis cells.

## Data Availability

Not applicable.
